# ROS-Induced Activation of DNA Damage Responses Drives Senescence-Like State in Postmitotic Cochlear Cells: Implication for Hearing Preservation

**DOI:** 10.1007/s12035-019-1493-6

**Published:** 2019-01-28

**Authors:** Nesrine Benkafadar, Florence François, Corentin Affortit, François Casas, Jean-Charles Ceccato, Julien Menardo, Frederic Venail, Bernard Malfroy-Camine, Jean-Luc Puel, Jing Wang

**Affiliations:** 1 INSERM - UMR 1051, Institut des Neurosciences de Montpellier, 80 rue Augustin Fliche, 34295 Montpellier, France; 2 Université Montpellier, 34295 Montpellier, France; 3 INRA, UMR 866 Différenciation Cellulaire et Croissance, 34060 Montpellier, France; 4 MindSet Rx, Arlington, MA USA

**Keywords:** Oxidative stress, DNA damage responses, Senescence, Age-related hearing loss, Hearing preservation

## Abstract

In our aging society, age-related hearing loss (ARHL) has become a major socioeconomic issue. Reactive oxygen species (ROS) may be one of the main causal factors of age-related cochlear cell degeneration. We examined whether ROS-induced DNA damage response drives cochlear cell senescence and contributes to ARHL from the cellular up to the system level. Our results revealed that sublethal concentrations of hydrogen peroxide (H_2_O_2_) exposure initiated a DNA damage response illustrated by increased γH2AX and 53BP1 expression and foci formation mainly in sensory hair cells, together with increased levels of p-Chk2 and p53. Interestingly, postmitotic cochlear cells exposed to H_2_O_2_ displayed key hallmarks of senescent cells, including dramatically increased levels of p21, p38, and p-p38 expression, concomitant with decreased p19 and BubR1 expression and positive senescence-associated β-galactosidase labeling. Importantly, the synthetic superoxide dismutase/catalase mimetic EUK-207 attenuated H_2_O_2_-induced DNA damage and senescence phenotypes in cochlear cells in vitro. Furthermore, systemic administration of EUK-207 reduced age-related loss of hearing and hair cell degeneration in senescence-accelerated mouse-prone 8 (SAMP8) mice. Altogether, these findings highlight that ROS-induced DNA damage responses drive cochlear cell senescence and contribute to accelerated ARHL. EUK-207 and likely other antioxidants with similar mechanisms of action could potentially postpone cochlear aging and prevent ARHL in humans.

## Introduction

Age-related hearing loss (ARHL) or presbycusis is the third most prevalent chronic disease among older adults [1]. It can vary in severity from mild to severe, the more severe forms affecting communication and contributing to social isolation, depression, and possibly dementia [2]. There is general agreement that cumulative effects of aging on hearing are exacerbated by genetic predisposition and environmental factors such as noise or drug exposure. With increasing noise pollution and life expectancy in our modern society, the prevalence of presbycusis is expected to grow dramatically. The precise mechanisms behind the age-related degeneration of the cochlear structures remain unclear, however. This is in part due to the complexity of each causal factor and to the interaction of the different mechanistic pathways leading to ARHL.

Growing evidence links oxidative stress to ARHL [3, 4]. The presence of lipid peroxidation [4], oxidative mitochondrial DNA damage, and glutathione-conjugated proteins [5, 6] is each strongly associated with cochlear aging in mice. In addition, reduced expression of antioxidant enzymes such as catalase, mitochondrial manganese superoxide dismutase (MnSOD or SOD2), and cytosolic SOD1 has been observed in older mice [4–6]. Furthermore, mice lacking the antioxidant enzyme SOD1 display enhanced age-related cochlear hair cell loss, reduced thickness of the stria vascularis, and severe degeneration of spiral ganglion neurons (SGNs) [7]. On the other hand, an increase in SOD2 expression gradient in ganglion cells has been reported along the basal to apical axis of rodent and primate cochlea [8], consistent with the differential vulnerability of the varying areas in the cochlea, from the most vulnerable high-frequency to least vulnerable low-frequency regions in most ARHL. Finally, low serum levels of the ROS scavenger melatonin are significantly associated with the occurrence of high-frequency hearing loss in the elderly [9]. Taken together, this experimental and clinical evidence indicates the potential involvement of oxidative stress in ARHL.

It is generally accepted that oxidative stress may cause irreversible DNA damage [10]. Over 100 oxidative modifications to DNA have been identified, including adducts and both single-strand and double-strand breaks [11]. In the cochlea, we have recently reported the activation of the DNA damage response (DDR) in postmitotic inner ear cells following treatment with the ototoxic anticancer drug cisplatin [12]. However, the link between increased ROS, DNA damage, and cochlear cell aging remains obscure. Understanding ROS-induced cochlear cell degeneration may lead to identifying key molecular targets to protect residual hearing in patients suffering ARHL.

The synthetic superoxide dismutase (SOD)/catalase mimetic EUK-207 is a member of the class of metal-containing salen–manganese (Mn) complexes. EUK-207 suppresses oxidative stress, including in the mitochondria [13, 14], through transforming oxygen free radicals into oxygen via a superoxide dismutase-like activity, and hydrogen peroxide into oxygen and water by a catalase-like activity [13]. EUK-207 has been used successfully to suppress age-related [15, 16] and radiation-induced cognitive impairment in mice [17].

Here, we explore the hypothesis that ROS-induced DDR drive cochlear cell senescence and contribute to ARHL from the cellular to the system level. In our in vitro study, hydrogen peroxide (H_2_O_2_) exposure caused DNA damage and initiated a DNA damage response in cochlear cells, as demonstrated by activation of the Chk2–p53 pathway. In addition, the H_2_O_2_-exposed cochlear cells exhibited several additional senescence-associated properties, including high senescence-associated β-galactosidase activity mainly in sensory hair cells, together with massively increased levels of p21, p38, and p-p38. Our in vivo results from senescence-accelerated mouse-prone 8 (SAMP8) mice, the well-characterized model of premature cochlear aging [4], demonstrated a similar accumulation of high amounts of DNA damage and similar senescence-like features in the cochlear tissues during aging. Importantly, EUK-207 attenuated H_2_O_2_-induced DNA damage and senescence phenotype in cochlear cells in vitro and reduced age-related loss of both hearing and hair cells in SAMP8 mice. These results highlight the attractive potential of antioxidants as a tool with which ARHL could be slowed down.

## Material and Methods

### Animals

Neonate Swiss mice were purchased from Janvier Laboratories. The senescence-accelerated mouse strain is derived from AKR/J mice by continuous brother–sister mating selected for a phenotype toward either accelerated senescence (SAMP) or resistance senescence (SAMR) [18]. Among these strains, SAMP8 mice have strain-specific characteristics of rapid aging [19]. Here, SAMP8 and senescence-accelerated mouse-resistant 1 (SAMR1) of both sexes were purchased from Harlan SARL Laboratory. Mice were housed in pathogen-free animal care facilities accredited by the French Ministry of Agriculture and Forestry (C-34-172-36; December 19, 2014).

### Drug Preparation

H_2_O_2_ was purchased from Acros Organics (#202460250). The salen–manganese (Mn) SOD-catalase mimetic, EUK-207, was a gift from Dr. Bernard Malfroy-Camine, of MindSet Rx, Arlington, MA, USA.

For in vitro experiments, H_2_O_2_ was freshly prepared in culture medium to final concentrations ranging from 0 to 1.25 mM. The EUK-207 was prepared at 10 mM in pure water and freshly diluted in culture medium to a final concentration of 10 μM. This final concentration was chosen based on our preliminary evaluations of the dose–response effects of EUK-207, at concentrations ranging from 0 to 30 μM, on H_2_O_2_-induced γH2AX expression on day 3 in H_2_O_2_-exposed cochlear explants. For in vivo experiments, EUK-207 was freshly prepared at 1.5 mM in 5% mannitol and was administered through a subcutaneously implanted Alzet micro-osmotic pump (DURECT Corporation, 0.11 μl per hour, 28 days #1004) at a dose of 0.2 mg/kg/day.

### In Vitro Assessments

#### Organ of Corti and Whole Cochlea Cultures

Mouse whole cochleae and organ of Corti explants were collected from postnatal day 3 mice and prepared according to the procedures described previously [12, 20]. The whole cochleae were kept in suspension, and the organ of Corti explants in adherent conditions in a 6-well culture plate containing 2 ml/well of culture medium. Culture medium consisted of Dulbecco’s modified Eagle’s medium/nutrient mixture F-12 (DMEM/F-12, #21331020) containing 2 mM L-glutamine (#25030024), N-2 complement (#17502048) at 1× and insulin transferrin selenium (#41400045) at 1× purchased from Gibco Life technologies, and 8.25 mM D-glucose (#G6152) and 30 U/ml penicillin G (#P3032) from Sigma-Aldrich.

#### H_2_O_2_-Induced Oxidative Stress

The oxidizing agent used to induce oxidative stress in vitro (H_2_O_2_) was chosen based on its wide use in a variety of cell types [21, 22]. Here, cochlear samples were firstly exposed to culture medium alone for 24 h in a humidified incubator (37 °C, 5% CO_2_). To probe the dose effect of H_2_O_2_, the culture medium was then replaced with fresh medium containing H_2_O_2_ at various concentrations (0, 0.25, 0.4, 0.5, 0.75, 1, and 1.25 mM) for 5 h before being replaced again with the culture medium alone and maintained for 5 days. The culture medium was renewed once on day 2 or 3 following its replacement. To determine time-dependent cochlear cell degeneration, organ of Corti explants were exposed to medium containing H_2_O_2_ at its half maximal effective concentration (EC_50_) and also at the concentration just below the EC_50_ (0.5 mM) for 5 h and then maintained in the culture medium alone for 0, 1, 3, and 5 days. All control samples were maintained in culture medium alone and were run concurrently alongside the experimental cultures.

#### Dose-Dependent Cytotoxicity and Apoptosis

To evaluate the toxicity of H_2_O_2_, cultured organ of Corti explants were immunolabeled with a rabbit polyclonal antibody against myosin 7A (1/300, Proteus Biosciences Inc. #25-6790) and a mouse monoclonal antibody against neurofilament (NF 200, 1/600, Sigma-Aldrich #N0142) to label hair cells and spiral ganglion neurons, respectively. Hair cell counting (six cochleae per condition and per time point) was performed using standard techniques [20]. Due to the resistance of apical turn hair cells to H_2_O_2_ cytotoxicity, counting concerned only a 1.5-mm length of the cochlear duct at the basal turn (4 to 5.5 mm from the apex, see [12]).

The TUNEL kit (DeadEnd™ fluorometric TUNEL System, Promega #G3250) was used to identify apoptotic DNA fragmentation. The cochlear samples were counterstained with a rabbit polyclonal antibody against myosin 7A and a mouse monoclonal antibody against neurofilament. All secondary antibodies were used at a dilution of 1/1000. This included donkey anti-mouse and anti-rabbit IgG conjugated to Alexa 488 or Alexa 568 (Molecular Probes #A-21202, #A-21206, #A-10037, #A-10042). Fluorescent tags were visualized using a confocal microscope (LSM 5 Live Duo, Zeiss). In control specimens without primary antibodies, neither Alexa 488 nor 568 fluorescent tags were observed. All experiments were performed in triplicate.

#### Measurement of Oxidative Stress

Oxidative stress was studied in the whole cochlea homogenates as previously described [4]. Catalase and SOD activities were measured as previously described by Marklund [23]. Lipid peroxidation was assessed using the thiobarbituric acid-reactive substances method and was expressed in nanomoles per milligram malondialdehyde (MDA) [24]. Protein concentrations were measured using the BCA protein assay kit (Pierce #23250). All experiments were performed in triplicate.

#### Cellular Localization of DNA Damage Foci and Foci Counting

Immunocytochemistry was employed to localize DNA damage foci in cultured organ of Corti using mouse monoclonal antibody against phospho-H2AX (1/500, Ser139, Merck Millipore #05-636) or rabbit polyclonal antibody against 53BP1 (1/100, Novus Biologicals #NB100-305). The cochlear samples were counterstained with a rabbit polyclonal antibody against myosin 7A or mouse monoclonal antibody against parvalbumin (1/500, SWANT #PV235) to label hair cells. All secondary antibodies were used at a dilution of 1/1000. The Hoechst 33342 dye (0.002% wt:vol in PBS 1×, Thermo Fisher Scientific #62249) was used to stain DNA. Fluorescent tags were visualized using a confocal microscope (LSM 5 Live Duo, Zeiss). No fluorescent signal was detected in the control specimens without primary antibodies.

The number of foci (i.e., γH2AX or 53BP1) per nucleus was computed using algorithms with Matlab custom-made software (MathWorks Company) that allow 3D rendering and visualization of “isosurfaces” enveloping all pixel clusters with intensities greater than a user-defined criterion value in each corresponding image channel (for more details, see [12]). All experiments were performed in triplicate.

#### Expression of Apoptosis, Oxidative Stress, and Autophagy-Related Proteins, DNA Damage Response, and Senescence Markers

Evaluation of apoptosis, oxidative stress, autophagy, DNA damage responses, and cellular senescence-like state in the cochlear protein extracts from H_2_O_2_-exposed and cultured whole cochleae was performed using Western blotting technique as described previously [4]. Antibodies used included those recognizing Bax (1/1000, Abcam #7977), BCl2 (1/1000, Santa Cruz #sc-492), cleaved caspase 3 (1/1000, Asp175 Cell Signaling #9661), SOD2 (1/1000, Abcam #ab13533), catalase (1/1000, Abcam #16731), p66^Shc^ (1/1000, Abcam #ab33770), phospho- p66^Shc^ (S36, 1/1000, Abcam #54518), p-Beclin 1 Ser93 (1/1000, Cell Signaling #14717), LC3-II (1/800, Cell Signaling #2775), Rab7 (1/800, Santa Cruz Biotechnology #sc-376,362), p62 (1/1000, MBL International #PM045), phospho-H2AX (1/1500, Ser139, Cell Signaling #2577), 53BP1 (1/1000, Novus Biologicals #100-305), DDB2 (1/800, Invitrogen #PA5-37361), phospho-Chk1 (1/1000, Ser345, Cell Signaling #2348), phospho-Chk2 (1/1000, Thr68, Cell Signaling #2661), p53 (1/1500, Cell Signaling #2524), p21 (1/2000, Cell Signaling #2946), p38 (1/500, Elabscience #E-AB-32459), p-p38 (Thr180/Tyr182, 1/1000, Cell Signaling #9211), p16 (1/1000, BD Pharmingen #551154), p19 (1/1000, Abcam #ab80), BubR1 (1/1000, Abcam #ab183496), FOXO3a (1/1000, Cell Signaling #2497), and nuclear factor erythroid 2–related factor 2 (Nrf2) (1/1000, Santa Cruz # sc-365949). β-Actin (1/10,000, Sigma-Aldrich #A1978) served as a loading control. Secondary antibodies used were horseradish peroxidase-conjugated goat anti-mouse IgG antibodies (1/3000, Jackson ImmunoResearch #115-001-003) or goat anti-rabbit IgG antibodies (1/3000, Jackson ImmunoResearch #111-001-003). All experiments were performed in triplicate. Image scans of Western blots were used for semiquantitative analysis.

#### Senescence-Associated Beta-Galactosidase Activity Assay

Senescence-associated beta-galactosidase (SA-β-gal) activity was measured according to the manufacturer’s protocol (Cell Signaling #9860). Briefly, cultured cochlear explants and cochleae from 6-month-old SAMR1 and SAMP8 mice were fixed in 4% paraformaldehyde in phosphate-buffered saline 1× (PBS) for 30 min at room temperature and washed twice with PBS. The fixed SAMR1 and SAMP8 cochleae were then processed for either cryosectioning or surface preparation. All cochlear samples were then incubated overnight at 37 °C in a dry incubator with fresh β-galactosidase staining solution at pH 6.0. Cochlear explants were observed using light microscopy, while cryosections and surface preparations were scanned using a NanoZoomer (Hamamatsu Photonics, Hamamatsu City, Japan) with a ×40 objective.

#### Pharmacological Mitigation of ROS

To probe the impact of ROS mitigation on H_2_O_2_-induced DNA damage and senescence-like phenotype, whole cochleae were exposed to either culture medium alone or medium containing 10 μM EUK-207 for 24 h in a humidified incubator (37 °C, 5% CO_2_). The culture medium was then replaced with fresh medium containing H_2_O_2_ at a concentration of 0.5 mM for 5 h. The cochlear samples were further grown for three additional days in culture medium either alone or with EUK-207. All control samples were maintained in culture medium and were run concurrently with experimental cultures. The samples were then collected for the evaluation of oxidative stress, DNA damage, and cellular senescence-like state using Western blotting and immunolabeling techniques.

### Validation in Adult SAMP8 In Vivo

Our previous study demonstrated that SAMP8 mice displayed premature ARHL and early onset cochlear cell degeneration when compared with the normally aging control senescence-accelerated mouse-resistant 1 (SAMR1) mice [4].

#### Functional Assessment

The auditory function was assessed by recording auditory brainstem responses (ABRs) in SAMP8 and SAMR1 mice at the age of 1, 3, 6, and 12 months (*n* = 14 animals per age and per strain). ABRs reflect the synchronous activation of auditory neurons from the cochleae up to the colliculi in response to incoming sound. The recordings were carried out under anesthesia with Rompun 2% (3 mg/kg) and Zoletil 50 (40 mg/kg) in a Faraday-shielded anechoic soundproof cage. Rectal temperature was measured with a thermistor probe and maintained at 38.5 ± 1 °C using a heated under blanket. ABRs were recorded from three subcutaneous needle electrodes placed at the vertex (active) and on the pinna of the tested ear, as well as in the neck muscles (ground) of the mice. The acoustic stimuli generated by a NI PXI-4461 signal generator (National Instruments) consisted of 10 ms tone bursts with a 1-ms rise and fall time delivered at a rate of 10/s. Sound was delivered by a JBL 075 loudspeaker (James B. Lansing Sound) positioned at 10 cm from the tested ear, under calibrated free-field conditions. Cochlear amplification (20,000) was achieved via a Grass P511 differential amplifier, averaged 1000 times (Dell Dimensions). Amplitude-intensity functions of the ABRs were obtained at each frequency tested (2, 4, 6.3, 8, 10, 12.5, 16, 20, 25, and 32 kHz) by varying the intensity of the tone bursts from 0 to 100 dB SPL, in 5 dB incremental steps. The ABR thresholds were defined as the minimum sound intensity necessary to elicit a well-defined and reproducible wave II. Recordings and analysis were performed blindly.

#### Molecular Assessment

Evaluation of oxidative stress, DNA damage responses, and cellular senescence-like state in the cochlear protein extracts from the cochleae of SAMP8 and SAMR1 mice aged 1, 6, and 12 months (*n* = 16 cochleae per age and per strain) was performed using Western blotting technique as described previously [4]. Antibodies used included those recognizing Nrf2 (1/1000, Santa Cruz #sc-365949), SOD2 (1/1000, Abcam #ab13533), p66^Shc^ (1/1000, Abcam #ab33770), phospho-p66^Shc^ (S36, 1/1000, Abcam #54518), phospho-Chk2 (1/1000, Thr68, Cell Signaling #2661), p53 (1/1500, Cell Signaling #2524), phospho-p53 (1/1500, Ser15, Cell Signaling #9289), p21 (1/2000, Cell Signaling #2946), p16 (1/1000, BD Pharmingen #551154), BubR1 (1/1000, Abcam #ab183496), and p19 (1/1000, Abcam #ab80). β-Actin (1/10,000, Sigma-Aldrich #A1978) served as a loading control. Secondary antibodies used were horseradish peroxidase-conjugated goat anti-mouse IgG antibodies (1/3000, Jackson ImmunoResearch #115-001-003) or goat anti-rabbit IgG antibodies (1/3000, Jackson ImmunoResearch #111-001-003). All experiments were performed in triplicate. Image scans of Western blots were used for semiquantitative analysis.

#### Age-Related Hearing Impairment and Molecular Correlation

To compare the time course of the hearing impairments found in SAMP8 and SAMR1 mice, the mean ABR threshold evoked by the range of tested frequencies from 2 to 32 kHz for each strain and at each time point was calculated. A linear regression was then performed to determine the threshold elevation per month. To decipher the molecular determinants of the accelerated age-related hearing impairment in SAMP8 mice, we used the mean values of ABR thresholds and protein levels in SAMR1 as a reference and subtracted these from the values for SAMP8 mice. The differences in threshold and protein level (SAMP8 − mean SAMR1) were then averaged and used for statistical analysis and to calculate SEM.

#### Pharmacological Counteraction of ROS

Finally, the impact of ROS mitigation on premature ARHL was also investigated in SAMP8 mice. The 6-month-old male and female SAMP8 mice were randomly divided into two groups: (i) control 5% mannitol (vehicle, *n* = 10) and (ii) EUK-207 (*n* = 10). The EUK-207 was dissolved in 5% mannitol to a final concentration of 1.5 mM, filter-sterilized, and administered at a dose of 0.2 mg/kg/day with the use of a subcutaneous Alzet micro-osmotic pump, continuing for up to 84 days. This dose of EUK-207 was selected according to the effective EUK-207 dose range reported previously [16]. Control mice received pumps filled with 5% mannitol. Mice received new pumps every month.

The ABRs were recorded prior to pump implantation and 2 and 3 months afterwards. Sensory hair cell loss was evaluated using scanning electron microscopy (Hitachi S4000). The cochleae from the different groups (*n* = 7 per group) were processed and evaluated using previously reported standard techniques [25, 26]. Hair cell counting was performed in four different 300-μm-long segments of the organ of Corti, centered at 1.1, 2.6, 3.5, or 4.1 mm from the cochlear apical end and corresponding to the frequencies of 8, 16, 25, and 32 kHz, respectively [27].

### Statistics

Data are expressed as mean ± SEM. Normality of the variables was tested by the Shapiro–Wilks test. The significance of the group differences was assessed with a one-way ANOVA; once the significance of the group differences (*P* < 0.05) was established, Tukey’s post hoc tests were subsequently used for pairwise comparisons. Significance of linear regressions was determined using the Pearson correlation coefficient (*P* < 0.001). For in vivo mouse studies, based on data from our previous reports [28, 29] or from preliminary experiments, we calculated the sample size using G*Power 3.1.9.2 to ensure adequate power of key experiments for detecting prespecified effect sizes.

## Results

### In Vitro Studies

To probe a direct causal relationship between increased ROS and premature occurrence of cochlear cell senescence-like phenotype, we adapted an available in vitro model of oxidative stress [30, 31], which consists of exposing cochlear explants or whole cochleae to a series of H_2_O_2_ concentrations in culture.

#### H_2_O_2_ Induces Dose-Dependent Outer Hair Cell Apoptosis

**Fig. 1:**
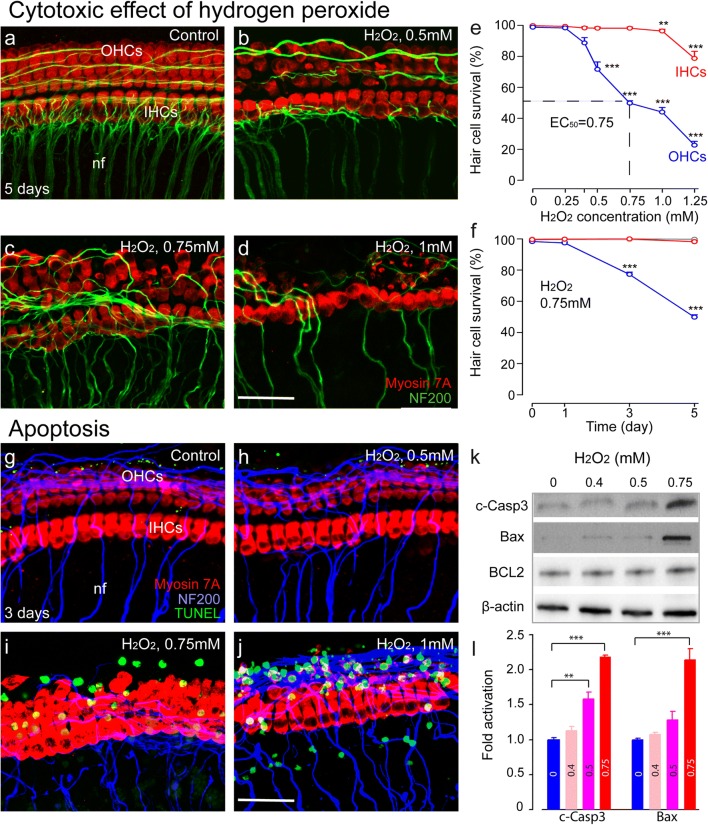
Hydrogen peroxide induces apoptosis of the cochlear outer hair cells in a dose and time dependent manner. **a**–**d** Confocal images showing the basal regions of cochlear explants treated with either culture medium alone (**a**) or medium containing 0.5 mM (**b**), 0.75 mM (**c**), or 1 mM (**d**) H_2_O_2_ for 5 h before being maintained in culture medium alone for 5 days. The explants were immunostained with myosin 7A (red) to identify hair cells and with neurofilament 200 (NF200, green) to visualize auditory nerve fibers. Scale bar = 20 μm. nf, auditory nerve fibers; OHCs, outer hair cells; IHCs, inner hair cells. **e** Dose–response curves of H_2_O_2_-induced loss of OHCs (blue line) and IHCs (red line) in basal cochlear regions. Data are expressed as mean ± SEM (*n* = 6 cochleae per condition). One-way ANOVA test followed by post hoc Tukey’s test (**P ≤ 0.01; ***P ≤ 0.001 vs. H_2_O_2_ 0 mM). **f** Effect over time on OHCs and IHCs of treatment with 0.75 mM H_2_O_2_ (blue and red lines for OHCs and IHCs, respectively) compared with culture medium alone (light blue and red lines for OHCs and IHCs, respectively). Data are expressed as mean ± SEM (*n* = 6 cochleae per condition and per time point). One-way ANOVA test was followed by post hoc Tukey’s test (***P ≤ 0.001 vs. H_2_O_2_ 0 mM). **g**–**j** Confocal images showing the basal region of cochlear explants treated with either culture medium alone (**g**) or containing 0.5 mM (**h**), 0.75 mM (**i**), or 1 mM (**j**) H_2_O_2_ for 5 h before their maintenance in culture medium alone for 3 days. Hair cells were identified using myosin 7A (red) and auditory nerve fibers with neurofilament 200 (NF200 blue). DNA fragmentation was identified using a TUNEL apoptosis kit (green). Scale bar = 35 μm. **k** Representative Western blot analysis using antibodies against cleaved caspase 3 (c-Casp3), Bax, BCL-2, and β-actin in whole cochlear extracts. **l** Histogram representing the fold change in cleaved caspase 3 and Bax expression levels in control and 0.4, 0.5, and 0.75 mM H_2_O_2_-exposed groups (*n* = 6 cochleae per condition). β-Actin served as a loading control. Data are expressed as mean ± SEM. One-way ANOVA test was followed by post hoc Tukey’s test (**P < 0.01, ***P < 0.001 vs. H_2_O_2_ 0 mM). All experiments were performed in triplicate

We firstly determined the survival of cochlear cells under increasing concentrations of H_2_O_2_. Our results show that a 5-h exposure to H_2_O_2_ in the millimolar range led to a significant concentration-dependent loss of outer hair cells (OHCs) (loss of OHCs—0.5 mM of H_2_O_2_: F1:22 = 14.2, *P* < 0.001; 0.75 mM: F1:34 = 986, *P* < 0.001; 1 mM: F1:27 = 552, *P* < 0.001; 1.25 mM: F1:34 = 884, *P* < 0.001) in the basal turn of the organ of Corti 5 days later, in contrast to an only slight loss of inner hair cells (IHCs) (loss of IHCs—1 mM: F1:20 = 13.72, *P* = 0.002; 1.25 mM: F1:27 = 41.93, *P* < 0.001) (Fig. 1a–e). A concentration of 1.25 mM provoked 77% of OHC and 20% of IHC loss (Fig. 1e). The counting of hair cells remaining after exposure to 0.75 mM H_2_O_2_ (EC_50_) and 0.5 mM (the concentration just below EC_50_) revealed a time-dependent OHC loss. Indeed, a concentration of 0.75 mM H_2_O_2_ led to a loss of 22.8 ± 1.4 and 50.2 ± 1.52% OHCs at 3 and 5 days after exposure, respectively (3 days: F1:34 = 14.2, *P* < 0.001, 5 days: F1:34 = 986, *P* < 0.001) (Fig. 1f), while 0.5 mM provoked a respective loss of only 11.6 ± 2 and 28.4 ± 4.7%. In contrast, IHCs showed only slight alterations up to 5 days after exposure at all concentrations. We therefore performed all molecular assessments within 3 days after H_2_O_2_ exposure.

To determine the nature of the H_2_O_2_-induced hair cell loss, we probed for apoptotic markers at 3 days after H_2_O_2_ exposure. Large amounts of OHCs and some IHCs displayed TUNEL-positive nuclei (Fig. 1i, j) after incubation with the higher H_2_O_2_ concentration (≥ 0.75 mM), when compared with controls or the sublethal concentration (0.5 mM, Fig. 1g, h). Concomitantly, Western blots from protein extracts of H_2_O_2_-exposed cochleae showed significant caspase 3 activation in the cochleae exposed to ≥ 0.5 mM of H_2_O_2_ (Fig. 1k, l) (0.5 mM of H_2_O_2_: F1:6 = 24.8, *P* = 0.003; 0.75 mM: F1:6 = 986, *P* < 0.001). In addition, higher levels of Bax protein were observed in those exposed to ≥ 0.75 mM of H_2_O_2_ (Fig. 1k, l) (F1:6 = 53.6, *P* < 0.001). In contrast, Bcl-2 protein levels remained unaltered (Fig. 1k). Taken together, these data demonstrate that H_2_O_2_ induces a concentration-dependent hair cell loss with massive OHC apoptosis after exposure to high concentrations (≥ 0.75 mM). In an in vivo situation, ROS levels are presumed very low due to their short half-life and the existence of various antioxidant enzymes [32]. On this basis, we chose H_2_O_2_ concentrations ≤ 0.5 mM for subsequent experiments.

#### Increased Oxidative Stress and Upregulation of Autophagy

**Fig. 2:**
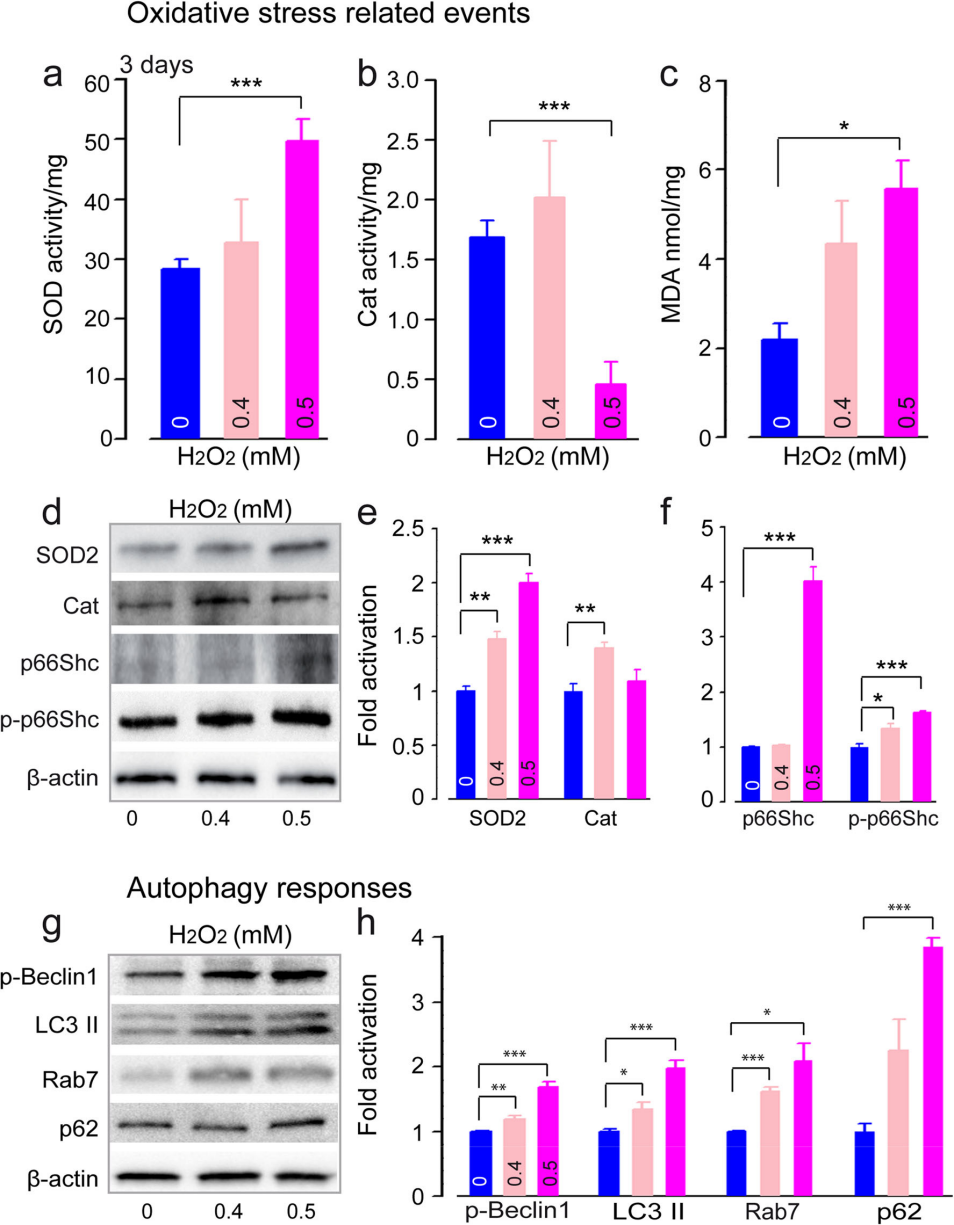
Increased oxidative stress and upregulation of autophagy. **a**–**c** Quantification analysis of superoxide dismutase (SOD) (**a**), catalase (Cat) (**b**), and malondialdehyde (MDA) (**c**) activities using spectrofluorochemistry in whole cochlear extracts from cochlea treated with culture medium alone (blue bars) or containing 0.4 mM (pale red bars) or 0.5 mM (pink bars) H_2_O_2_ for 5 h. The cochleae were collected from the different conditions after 3 days of culture. All the data are expressed as mean ± SEM (*n* = 16 cochleae per condition). One-way ANOVA test was followed by post hoc Tukey’s test (*P ≤ 0.04, ***P ≤ 0.001 vs. H_2_O_2_ 0 mM). **d** Representative Western blot analysis using antibodies against SOD2, Cat, p66^Shc^, p-p66^Shc^, and β-actin in whole cochlear extracts. **e**, **f** Histogram representing the levels of SOD2, Cat, p66^Shc^, and p-p66^Shc^ in control and 0.4 and 0.5 mM H_2_O_2_-exposed groups (*n* = 6 cochleae per condition). β-Actin served as a loading control. Data are expressed as mean ± SEM. One-way ANOVA test followed by post hoc Tukey’s test (*P ≤ 0.04, **P ≤ 0.01, ***P ≤ 0.001 vs. H_2_O_2_ 0 mM). **g** Representative Western blot analysis using antibodies against p-Beclin1, LC3-II, Rab7, p62, and β-actin in whole cochlear extracts. **h** Histograms representing the levels of p-Beclin1, LC3-II, Rab7, and p62 in control and 0.4 and 0.5 mM H_2_O_2_-exposed groups (*n* = 6 cochleae per condition). β-Actin served as a loading control. Data are expressed as mean ± SEM. One-way ANOVA test followed by post hoc Tukey’s test (*P ≤ 0.04, **P ≤ 0.01, ***P ≤ 0.001 vs. H_2_O_2_ 0 mM). All experiments were performed in triplicate

To confirm the occurrence of oxidative stress in cochlear tissues after exposure to H_2_O_2_ at concentrations ≤ 0.5 mM, we assessed the activity of antioxidant enzymes such as SOD and catalase (Cat), lipid peroxidation, and the expression levels of SOD2, Cat, p66^Shc^ and serine 36-phosphorylated p66^Shc^. P66^Shc^ is a redox protein playing an important role in the regulation of the cellular response to oxidative stress [33]. Three days after H_2_O_2_ exposure, cochleae treated with 0.5 mM H_2_O_2_ showed significantly increased SOD activity (Fig. 2a) (F1:12 = 28.6, *P* < 0.001) and lipid peroxidation (MDA, Fig. 2c) (F1:12 = 6.1, *P* = 0.03), but significantly decreased catalase activity (Fig. 2b) (F1:12 = 27.6, *P* < 0.001). Western blot analysis revealed significantly higher levels of SOD2 (0.4 mM of H_2_O_2_: F1:6 = 25.9, *P* = 0.002; 0.5 mM: F1:6 = 194.9, *P* < 0.001), Cat (0.4 mM: F1:6 = 24, *P* = 0.003), p66^Shc^ (0.5 mM: F1:6 = 253, *P* < 0.001), and p-p66^Shc^ (0.4 mM: F1:6 = 8.5, *P* = 0.027; 0.5 mM: F1:6 = 109, *P* < 0.001) in the cochleae exposed to H_2_O_2_ when compared with control-cultured cochleae (Fig. 2d–f). Collectively, these results show that events related to oxidative stress occur after exposure to H_2_O_2_ at low concentrations.

Considering the link between oxidative stress and autophagy [34], we examined autophagy induction by monitoring the levels of proteins involved in autophagosome formation and autophagic flux, the dynamic process of autophagosome synthesis, and degradation. H_2_O_2_-exposed cochleae displayed significantly increased levels of phosphorylated Beclin 1, a major player in the autophagic initiation process [35] (0.4 mM: F1:6 = 24.7, *P* = 0.003; 0.5 mM: F1:6 = 134.1, *P* < 0.001); LC3-II (0.4 mM: F1:6 = 10.3, *P* = 0.018; 0.5 mM: F1:6 = 53.7, *P* < 0.001), a hallmark of autophagosome formation; and Rab7, a small GTP-binding protein that plays a role in maturation of late autophagic vacuoles [36] (0.4 mM: F1:6 = 57.4, *P* < 0.001; 0.5 mM: F1:6 = 10.2, *P* = 0.029) (Fig. 2g, h). Identically, a significant increase of p62, a ubiquitin-binding scaffold protein and a subtract of autophagy, was observed in 0.5 mM H_2_O_2_-exposed cochleae (F1:6 = 255, *P* < 0.001) (Fig. 2g, h). Taken together, these results indicate that H_2_O_2_ induced cochlear cell oxidative stress and triggered an autophagic response.

#### DNA Damage Responses

**Fig. 3:**
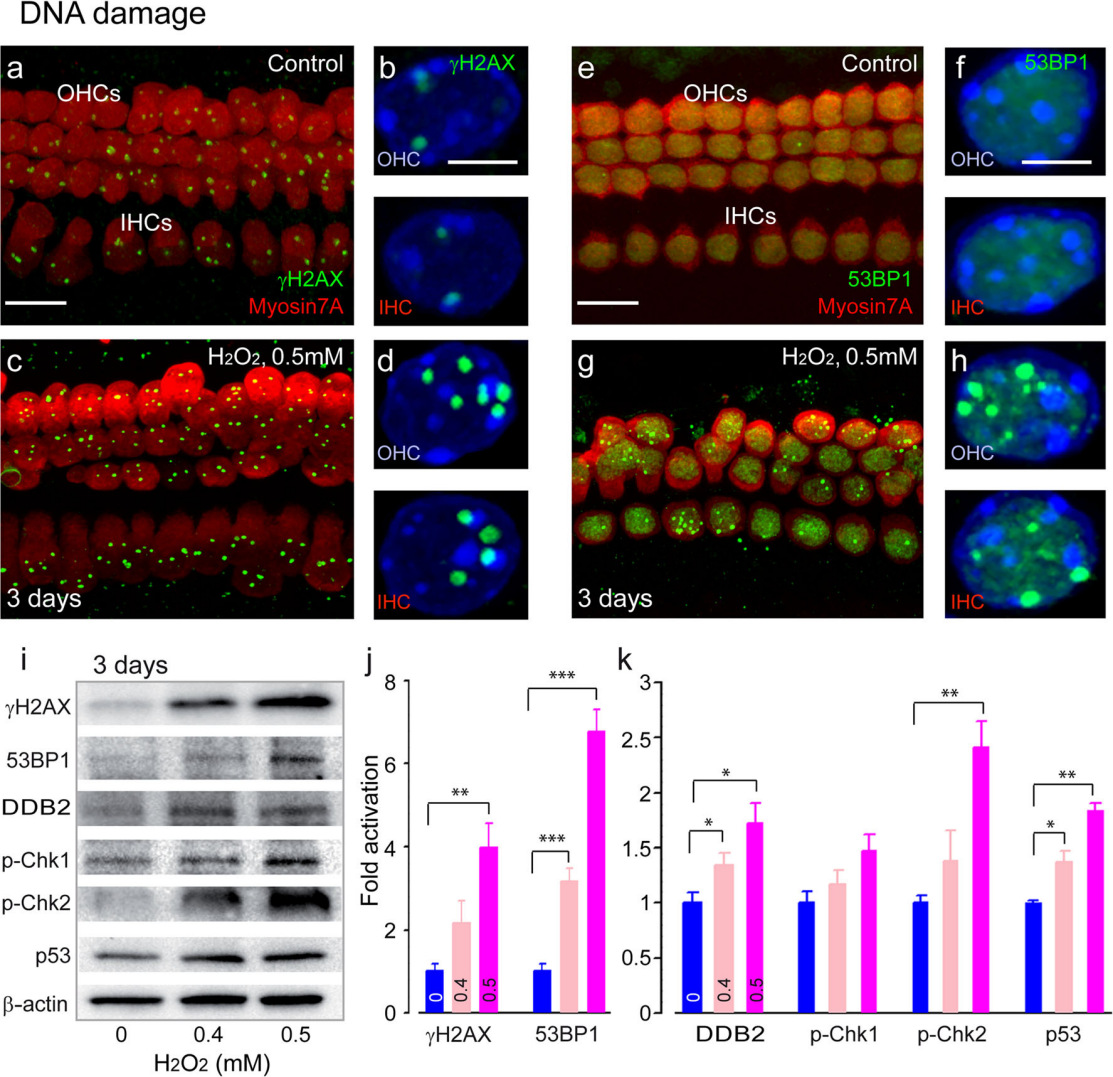
DNA damage and DNA damage responses upon H_2_O_2_ challenge. **a**, **c**, **e**, **g** Confocal images showing the basal region of the organ of Corti cultures treated with either culture medium alone (**a**, **e**) or containing 0.5 mM H_2_O_2_ (**c**, **g**) for 5 h before being maintained in culture medium alone for 3 days. The samples were then immunolabeled for myosin 7A (red, **a**, **c**, **e**, **g**), γH2AX (green, **a** and **c**) and 53BP1 (green, **e** and **g**). Scale bars: **a**, **c**, **e** and **g** = 10 μm. **b**, **d**, **f**, **h** Higher magnification images of representative OHC and IHC nuclei from all conditions tested. Scale bar = 2.5 μm. **i** Representative Western blot analysis using antibodies against γH2AX, 53BP1, DDB2, p-Chk1, p-Chk2, p53, and β-actin in whole cochlear extracts. **j**, **k** Histograms representing the levels of γH2AX, 53BP1, DDB2, p-Chk1, p-Chk2, and p53 in control and in 0.4 and 0.5 mM H_2_O_2_-exposed groups (*n* = 6 cochleae per condition). β-Actin served as a loading control. Data are expressed as mean ± SEM. One-way ANOVA test followed by post hoc Tukey’s test (*P ≤ 0.04, **P ≤ 0.01, ***P ≤ 0.001 vs. H_2_O_2_ 0 mM). All experiments were performed in triplicate

To determine the nature of DNA damage induced by H_2_O_2_ in hair cell nuclei, we used immunolabeling to detect and localize H2AX phosphorylation (γH2AX) and 53BP1, two hallmarks of DNA damage. H_2_O_2_-intoxicated organ of Corti (Fig. 3c, d) exhibited significantly higher levels of γH2AX foci formation in both outer hair cells (4.61 ± 0.55 foci per nucleus) and inner hair cells (4.21 ± 0.22 per nucleus) when compared to the control organ of Corti cultures (Fig. 3a, b, 3.22 ± 0.18 vs. 2.88 ± 0.27 foci per nucleus for OHC and IHC, respectively) (OHCs: F1:42 = 98.2, *P* < 0.001; IHCs: F1:34 = 286, *P* < 0.001). While the control organ of Corti cultures displayed almost no 53BP1 foci indicative of DNA double-strand breaks [37, 38] (Fig. 3e, f), the 0.5-mM H_2_O_2_-intoxicated cochleae showed a large number of 53BP1 foci (4.27 ± 1.27 and 4.25 ± 0.56 foci per nucleus for IHC and OHC, respectively) (Fig. 3g, h). Concomitantly, Western blots from protein extracts of H_2_O_2_-exposed cochleae revealed strong expression of both γH2AX and 53BP1 when compared with control-cultured cochleae (Fig. 3i, j) (γH2AX—0.5 mM of H_2_O_2_: F1:6 = 23.6, *P* = 0.003; 53BP1—0.4 mM of H_2_O_2_: F1:6 = 67.9, *P* < 0.001, 0.5 mM: F1:6 = 205.9, *P* < 0.001). Altogether, these results indicate the occurrence of DNA damage, including DNA double-strand breaks in H_2_O_2_-intoxicated cochleae.

DNA double-strand breaks are potent inducers of DDR, characterized by activation of DDR proteins, including the cell cycle checkpoint proteins like checkpoint kinase 1 (p-Chk1), checkpoint kinase 2 (p-Chk1), and p53 [39]. Once activated, p53 plays an important role in modulating distinct cell fate decisions [40–42]. It enhances DNA repair pathways through the upregulation of several repair proteins like the damage-specific DNA binding protein 2 (DDB2), which is required for nucleotide excision repair [43]. We found significant increases in the levels of DDB2 (0.4 mM of H_2_O_2_: F1:6 = 6.0, *P* = 0.049; 0.5 mM: F1:6 = 12.1, *P* = 0.013), p-Chk2 (0.5 mM: F1:6 = 30, *P* = 0.002), and p53 (0.4 mM: F1:6 = 9.2, *P* = 0.023; 0.5 mM: F1:6 = 25.8, *P* = 0.002) in H_2_O_2_-exposed cochleae when compared with the control condition (Fig. 3i, k). In contrast, the increase in the level of p-Chk1 after H_2_O_2_ challenge did not reach significance (Fig. 3i, k). These results indicate that H_2_O_2_ exposure induces DNA damage and the activation of the Chk2–p53 pathway in postmitotic cochlear cells.

#### Senescence-Like Phenotype in Postmitotic Cochlear Cells

**Fig. 4:**
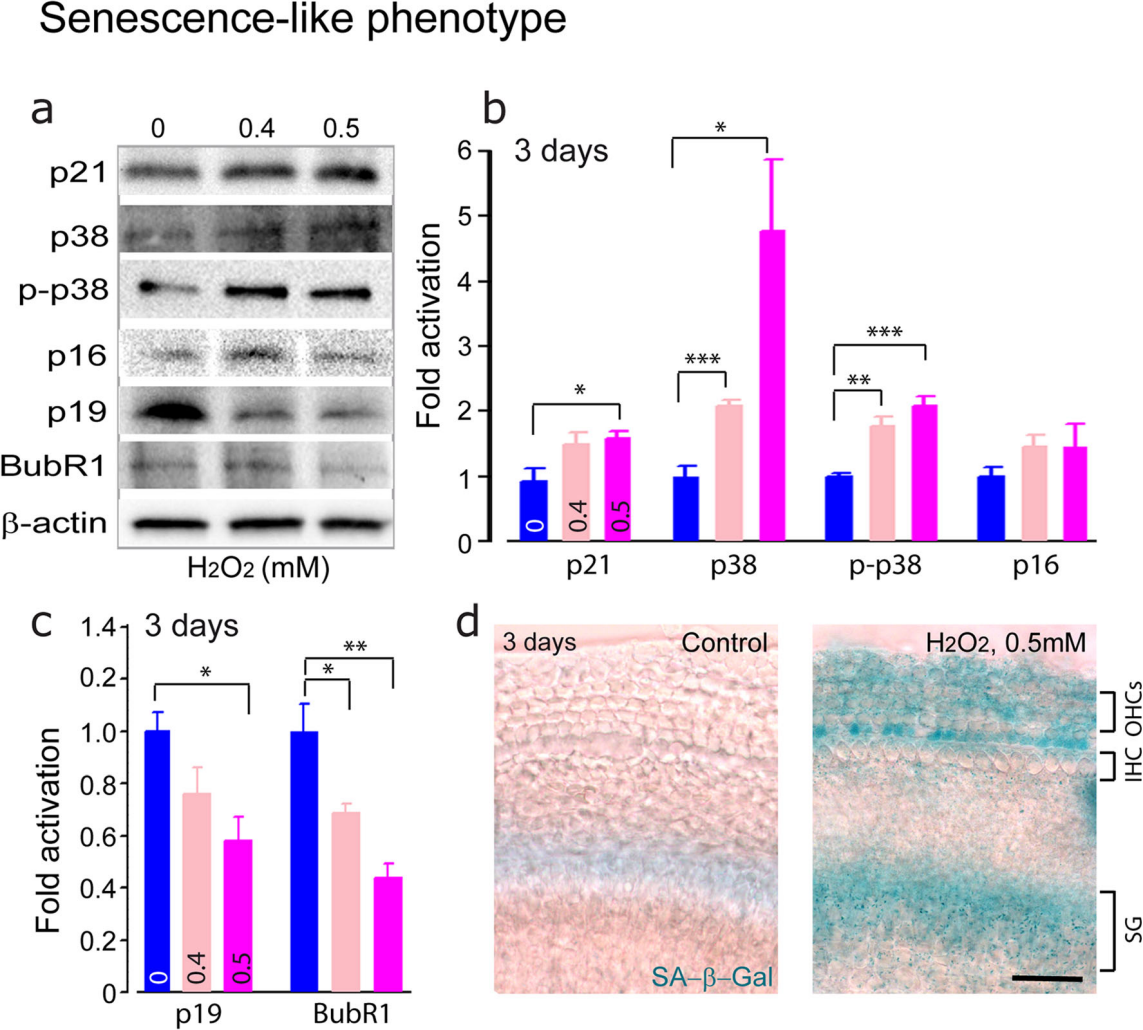
Senescence-like phenotype upon H_2_O_2_ treatment. **a** Representative Western blot analysis using antibodies against p21, p38, p-p38, p16, p19, BubR1, and β-actin in whole cochlear extracts. **b**, **c** Histograms representing the levels of p21, p38, p-p38, p16, p19, and BubR1 in 0 (control), 0.4, and 0.5 mM H_2_O_2_-exposed groups (*n* = 6 cochleae per condition). β-Actin served as a loading control. Data are expressed as mean ± SEM. One-way ANOVA test followed by post hoc Tukey’s test (*P ≤ 0.05, **P ≤ 0.01, ***P ≤ 0.001 vs. H_2_O_2_ 0 mM). **d** Light microscope images showing the basal region of the organ of Corti cultures treated with either culture medium alone or containing 0.5 mM H_2_O_2_ for 5 h before their maintenance in culture medium alone for 3 days. The samples were then stained with fresh β-galactosidase (SA-β-gal) solution at pH 6.0. Note that H_2_O_2_ exposure resulted in an increased activity of SA-β-gal (blue) in OHCs and supporting cells located in the area of the organ of Corti as well as in the cells located in the region of the spiral ganglion (SG). Scale bar = 25 μm. All experiments were performed in triplicate

To assess whether DDR induces a senescence-like phenotype in postmitotic cochlear cells in vitro*,* we examined the accumulation of p21, one of the downstream effectors of p53. We showed that 0.5 mM H_2_O_2_ resulted in a significant accumulation of p21 (Fig. 4a, b) (F1:6 = 7.9, *P* = 0.031), suggesting the activation of p53–p21 pathway. Considering both p21-dependent and p21-independent p38MAPK activation may occur in senescence [44], we probed the expression level of p38 and its phosphorylation on threonine-180 and tyrosine-182. Consistent with the activation of p53–p21, H_2_O_2_-exposed cochleae showed increased levels of p38 (0.4 mM: F1:6 = 35.4, *P* = 0.001; 0.5 mM: F1:6 = 11.3, *P* = 0.015) and phospho-p38 (0.4 mM: F1:6 = 27.8, *P* = 0.002; 0.5 mM: F1:6 = 52.3, *P* < 0.001) (Fig. 4a, b). H_2_O_2_ exposure did not significantly affect the expression level of p16, a cell cycle inhibitor. However, the cell cycle regulator p19 (0.5 mM of H_2_O_2_: F1:6 = 13.4, *P* = 0.011) and the mitotic checkpoint protein BubR1 (0.4 mM: F1:6 = 9.8, *P* = 0.02; 0.5 mM: F1:6 = 27, *P* = 0.002) were significantly repressed (Fig. 4a, c). Concomitantly, H_2_O_2_-exposed cochleae showed increased activity of SA-β-gal, a widely accepted general marker of the senescent phenotype [45] (Fig. 4d). Altogether, these data firmly demonstrate the occurrence of a senescence-like phenotype in postmitotic cochlear cells after H_2_O_2_ challenge.

#### Pharmacological Mitigation of ROS with EUK-207

**Fig. 5:**
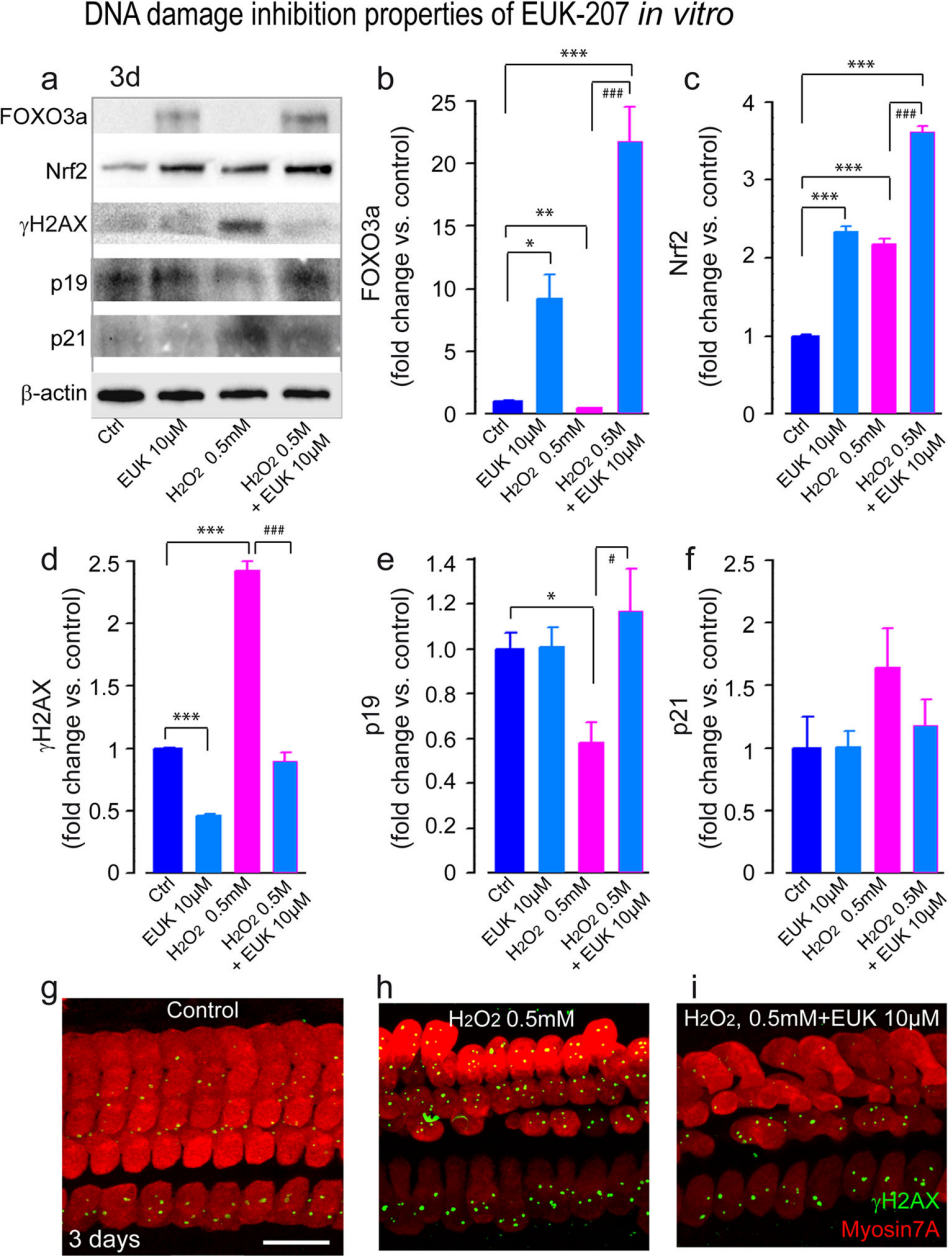
EUK-207 upregulates antioxidant response elements and suppresses DNA damage and senescence phenotype. **a** Representative Western blot analysis using antibodies against FOXO3a, Nrf2, γH2AX, p19, p21, and β-actin in whole cochlear extracts. **b**–**f** Histograms representing the levels of FOXO3a, Nrf2, γH2AX, p19, and p21 in control (Ctrl), 10 μM EUK-207 alone, and 0.5 mM H_2_O_2_ alone or in combination with 10 μM EUK-207 exposed groups (*n* = 6 cochleae per condition). β-Actin served as a loading control. Data are expressed as mean ± SEM. One-way ANOVA test followed by post hoc Tukey’s test (*P ≤ 0.04, **P ≤ 0.01, ***P ≤ 0.001 vs. Ctrl; ^**#**^*P* ≤ 0.025, ^**##**^*P* ≤ 0.01, ^**###**^*P* ≤ 0.001 vs. H_2_O_2_ 0.5 mM). **g**–**i** Confocal images showing the basal region of organ of Corti cultures treated with either culture medium alone (control, **g**) or with medium containing 0.5 mM H_2_O_2_ (**h**) or 0.5 mM H_2_O_2_ in combination with 10 μM EUK-207 (**i**) for 3 days. The samples were then immunolabeled for myosin 7A (red) and γH2AX (green). Scale bar = 15 μm. All experiments were performed in triplicate

To probe the impact of ROS mitigation on H_2_O_2_-induced DNA damage and senescence-like phenotype, we aimed to counteract excessive ROS by pre- and posttreating cochleae with 10 μM EUK-207, a potent synthetic superoxide dismutase/catalase mimetic that scavenges superoxide and hydrogen peroxide [46]. As a readout, we first analyzed the expression of Foxo3a, a forkhead transcription factor of the Foxo class, which is known to reduce the level of cellular oxidative stress by directly increasing mRNA and protein levels of MnSOD and catalase [47]. We found that treatment with EUK-207 alone significantly increased the levels of Foxo3a (Fig. 5a, b) (F1:6 = 8.9, *P* = 0.029). We also established that EUK-207 alone increased the Nrf2 levels (Fig. 5a, c) (F1:6 = 157.2, *P* < 0.001), but significantly reduced γH2AX levels (Fig. 5a, d) (F1:6 = 65.2, *P* < 0.001). Interestingly, a combined treatment with 10 μM EUK-207 and 0.5 mM H_2_O_2_ significantly increased the expression levels of Foxo3a and Nrf2 when compared with the control (Foxo3a: F1:6 = 118.2, *P* < 0.001; Nrf2: F1:6 = 751.2, *P* < 0.001) or H_2_O_2_ alone (Foxo3a: F1:6 = 124.8, *P* < 0.001; Nrf2: F1:6 = 221.2, *P* < 0.001) (Fig. 5a–c). In contrast, this combination efficiently attenuated the induction of γH2AX by H_2_O_2_ (Fig. 5a, d, h–i) (F1:6 = 174.8, *P* < 0.001). Finally, we also report that this combined treatment significantly increased p19 expression (Fig. 5a, e) (F1:6 = 8.9, *P* = 0.025), but did not significantly reduce p21 level (Fig. 5a, f). Taken together, these results demonstrate that by counteracting excessive ROS, EUK-207 may attenuate oxidative stress-induced DNA damage and senescence phenotype.

### In Vivo Validation in Adult SAMP8 Mice

#### Molecular Pathway Validation

**Fig. 6:**
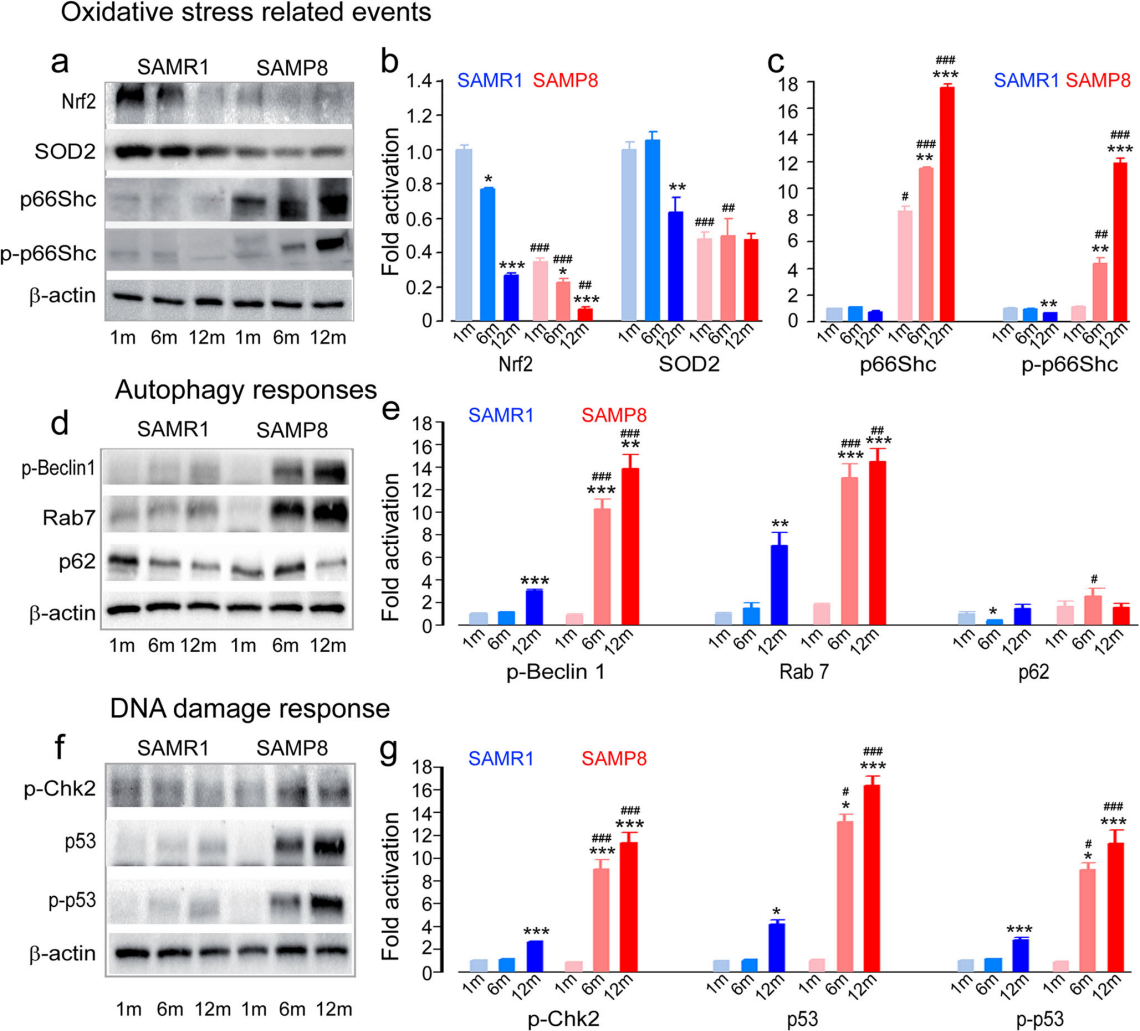
Increased oxidative stress, upregulation of autophagy, and activation of DDR in adult SAMP8. **a**, **d**, **f** Representative Western blot analysis using antibodies against Nrf2, SOD2, p66^Shc^, p-p66^Shc^, p-Beclin1, Rab7 p62, p-Chk2, p53, p-p53, and β-actin in whole cochlear extracts. **b**, **c** Histograms representing the levels of Nrf2, SOD2, p66^Shc^, and p-p66^Shc^ in SAMR1 and SAMP8 mice aged 1, 6, and 12 months (*n* = 16 cochleae per strain and per age) (*P ≤ 0.05, **P = 0.01, ***P ≤ 0.001 vs. 1 month age; ^#^*P* ≤ 0.042, ^##^*P* ≤ 0.01, ^###^*P* ≤ 0.001 vs. SAMR1 of the same age). **e** Histograms representing the levels of p-Beclin1, Rab7, and p62 in SAMR1 and SAMP8 mice aged 1, 6, and 12 months (*n* = 16 cochleae per strain and per age) (*P = 0.024, **P ≤ 0.01, ***P ≤ 0.001 vs. 1 month age; ^#^*P* = 0.022, ^##^*P* = 0.0036, ^###^*P* ≤ 0.001 vs. SAMR1 of the same age). **g** Histograms representing the levels of p-Chk2, p53, and p-p53 in SAMR1 and SAMP8 mice aged 1, 6, and 12 months (*n* = 16 cochleae per strain and per age) (*P ≤ 0.043, **P ≤ 0.01, ***P ≤ 0.001 vs. 1 month age; ^#^*P* ≤ 0.025, ^##^*P* ≤ 0.01, ^###^*P* ≤ 0.001 vs. SAMR1 of the same age). β-Actin served as a loading control. Data are expressed as mean ± SEM. One-way ANOVA test followed by post hoc Tukey’s test. All experiments were performed in triplicate

SAMP8 mice have been shown to display premature hearing loss and cochlear degeneration associated with oxidative stress and altered levels of antioxidant enzymes [4]. Here, we probed whether cochlear cells engender similar DNA damage responses and senescence signatures during premature aging in vivo. We first analyzed Nrf2, emerging as a regulator of cellular resistance to oxidants [48]. Western blot analyses revealed a significant decrease in Nrf2 with age in both strains (Fig. 6a, b) (SAMR1: 6 months: F1:6 = 9.7, *P* = 0.021, 12 months: F1:6 = 92.9, *P* < 0.001; SAMP8: 6 months: F1:6 = 7.9, *P* = 0.031, 12 months: F1:6 = 78.2, *P* < 0.001). However, SAMP8 mice systematically showed significantly lower expression levels compared to the control SAMR1 mouse strains at all ages analyzed (Fig. 6a, b) (1 month: F1:6 = 104.5, *P* < 0.001; 6 months: F1:6 = 82.6, *P* < 0.001; 12 months: F1:6 = 14.6, *P =* 0.009). Interestingly, this much decreased Nfr2 expression was detectable from 1 month of age in SAMP8 mice (Fig. 6a, b). Similarly, while SAMP8 mice displayed a significantly lower level of MnSOD (SOD2) expression starting early from 1 month of age and maintained to 12 months (Fig. 6a, b) (1 month: F1:10 = 69.6, *P* < 0.001; 6 months: F1:10 = 17.6, *P* = 0.002), SAMR1 mice only showed such decrease in SOD2 at 12 months of age (Fig. 6a, b) (F1:10 = 14.6, *P* = 0.003). SAMP8 mice showed significantly higher levels of p66^Shc^ (1 month: F1:6 = 10.4, *P* = 0.029; 6 months: F1:6 = 657, *P* < 0.001; 12 months: F1:6 = 598.7, *P* < 0.001) and phorphorylated p66^Shc^ (p-p66^Shc^, 6 months: F1:6 = 24.1, *P* = 0.003; 12 months: F1:6 = 136.6, *P* < 0.001) when compared with SAMR1 mice of the same age (Fig. 6a, c). They also showed dramatic increases in the amounts of p66^Shc^ (6 months: F1:6 = 19.7, *P* = 0.004; 12 months: F1:6 = 108.9, *P* < 0.001) and p-p66^Shc^ (6 months: F1:6 = 21.9, *P* = 0.003; 12 months: F1:6 = 125.8, *P* < 0.001) during the aging process (Fig. 6a, c). In contrast, SAMR1 mice showed reduced levels of p-p66^Shc^ at 12 months compared to 1 month of age (Fig. 6a, c; F1:6 = 19.5, *P* = 0.004). Taken together, these results indicate the earlier occurrence (1 month old) and a higher level of oxidative stress in the cochleae of SAMP8 mice.

As expected, SAMP8 mice displayed a significant increase in phosphorylated Beclin 1 (6 months: F1:6 = 114.8, *P* < 0.0001; 12 months: F1:6 = 78, *P* = 0.006) and Rab7 (6 months: F1:6 = 82, *P* = 0.0001; 12 months: F1:6 = 85.3, *P* = 0.0001) from 6 months of age. In contrast, SAMR1 mice only showed such increases (p-Beclin 1: F1:6 = 167, *P* < 0.0001; Rab7: F1:6 = 26.3, *P* = 0.0022) at 12 months of age. SAMP8 mice also showed significantly higher levels of phosphorylated Beclin 1 (6 months: F1:6 = 108, *P* < 0.0001; 12 months: F1:6 = 73.3, *P* = 0.0001) and Rab7 (6 months: F1:6 = 76, *P* = 0.0001; 12 months: F1:6 = 21.2, *P* = 0.0036) than SAMR1 mouse strains at the same age (Fig. 6d, e). Not surprisingly, a significant higher level of p62 was observed in SAMP8 mice at 6 months compared with SAMR1 of the same age (F1:6 = 9.48, *P* = 0.022). While p62 levels maintained in SAMP8 mice during aging, these levels decreased significantly in SAMR1 mice at 6 months when compared with 1 month (F1:6 = 8.98, *P* = 0.0241)*.* Taken together, these results indicate an increased autophagy with impaired autophagic flux in SAMP8 mice.

To further explore the potential appearance of DDR in SAMP8 mice, we assessed DDR proteins. Western blot analysis demonstrated dramatic increases in p-Chk2 levels from as early as 6 months in SAMP8 mouse cochleae (6 months: F1:6 = 161.9, *P* < 0.001; 12 months: F1:6 = 292, *P* < 0.001), while such an increase was only seen in 12-month-old SAMR1 mice (F1:6 = 209.5, *P* < 0.001) compared to 1-month olds (Fig. 6f, g). In addition, significantly higher levels of p-Chk2 were observed in SAMP8 than SAMR1 mice of the same age (6 months: F1:6 = 151.4, *P* < 0.001; 12 months: F1:6 = 199, *P* < 0.001) (Fig. 6f, g). Similarly, SAMP8 mice showed significantly increased p53 (6 months: F1:6 = 11.2, *P* = 0.029; 12 months: F1:6 = 358.6, *P* < 0.001) and p-p53 levels (6 months: F1:6 = 11.1, *P* = 0.029; 12 months: F1:6 = 129.8, *P* < 0.001) from 6 months (Fig. 6f, g). The same phenomenon occurred in SAMR1 but at a later stage (at 12 months, p53: F1:6 = 10.9, *P* = 0.029; p-p53: F1:6 = 70.7, *P* < 0.001, Fig. 6f, g). However, changes were more pronounced in SAMP8 than in SAMR1 mice (p53: 6 months: F1:6 = 11, *P* = 0. 029, 12 months: F1:6 = 181.6, *P* < 0.001; p-p53: 6 months: F1:6 = 11.2, *P* = 0.029, 12 months: F1:6 = 84.1.2, *P* < 0.001, Fig. 6f, g). Together, these results point toward the activation of a DNA damage response during cochlear aging, with more severe effects in SAMP8 compared to SAMR1 mice.

**Fig. 7:**
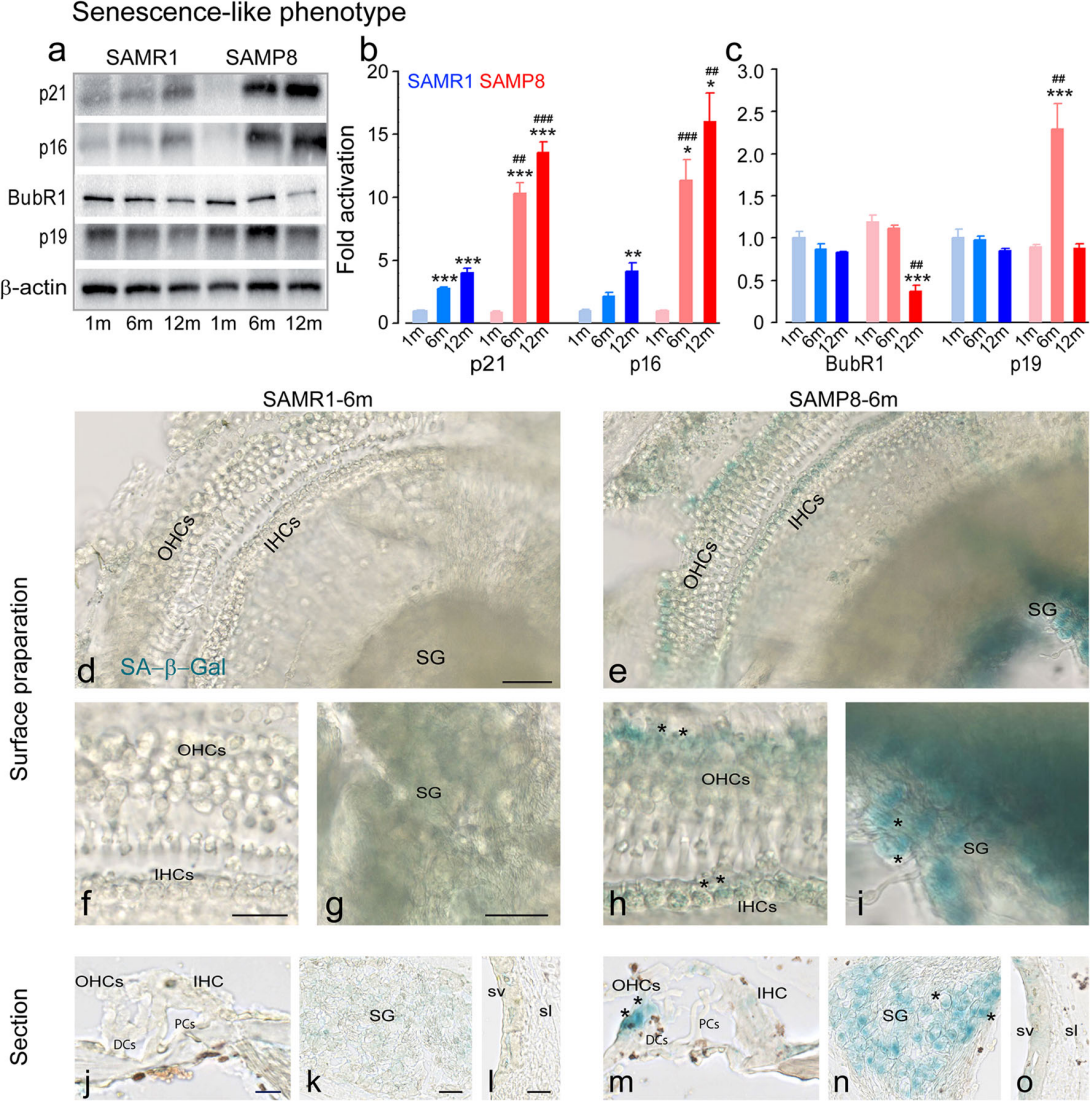
Senescence-like phenotype in adult SAMP8. **a** Representative Western blot analysis using antibodies against p21, p16, BubR1, p19, and β-actin in whole cochlear extracts. **b**, **c** Histograms representing the levels of p21, p16, BubR1, p19, and β-actin in SAMR1 and SAMP8 mice aged 1, 6, and 12 months (*n* = 16 cochleae per strain and per age). β-Actin served as a loading control. Data are expressed as mean ± SEM. One-way ANOVA test was followed by post hoc Tukey’s test (*P ≤ 0.035, **P = 0.01, ***P = 0.001 vs. 1 month age; ^#^*P* ≤ 0.041, ^##^*P* ≤ 0.01, ^###^*P* ≤ 0.001 vs. SAMR1 of the same age). All experiments were performed in triplicate. **d**, **e** Representative scanned images of cochlear surface preparations from the middle turn of the cochleae of SAMR1 (**d**) and SAMP8 (**e**) mice at 6 months. The samples were stained with fresh SA-β-gal solution at pH 6.0. Scale bar = 50 μm. **f**–**i** Higher magnification images of representative organ of Corti (**f**, **h**) and spiral ganglion (**g**, **i**) derived from **d** and **e**. Scale bars = 20 μm. **j**–**o** Representative scanned images of transverse cryostat sections of the organ of Corti (**j**, **m**), spiral ganglion (**k**, **n**), and stria vascularis (**l**, **o**) from SAMR1 (**j**–**l**) and SAMP8 (**m**–**o**) mice at 6 months. Scale bars: **j**, **k**, **m**, **n** = 10 μm; **l**, **o** = 25 μm. Note that SA-β-gal (blue)-stained cells are mostly present in the region of spiral ganglion neurons, OHCs, and IHCs of SAMP8 mice (asterisks in **h**, **i**, **m**, **n**). SG, spiral ganglion; DCs, Deiters cells; PCs, pillar cells; sv, stria vascularis; sl, spiral ligament

In addition, both strains displayed an increase in senescence-like features, characterized by an increase in the levels of p21 (SAMP8: 6 months: F1:6 = 44.4, *P* < 0.001, 12 months: F1:6 = 84.3, *P* < 0.001; SAMR1: 6 months: F1:6 = 151.8, *P* < 0.001, 12 months: F1:6 = 78.1, *P* < 0.001, Fig. 7a, b) and p16 during aging (SAMP8: 6 months: F1:6 = 11, *P* = 0.03, 12 months: F1:6 = 10.1, *P* = 0.029; SAMR1: 12 months: F1:6 = 14.6, *P* = 0.009, Fig. 7a, b). However, here again, changes were more pronounced in SAMP8 than in SAMR1 mice (p21: 6 months: F1:6 = 28.5, *P* = 0.002, 12 months: F1:6 = 46, *P* < 0.001; p16: 6 months: F1:6 = 31.9, *P* < 0.001, 12 months: F1:6 = 23.2, *P* = 0.003, Fig. 7a, b). SAMP8 mice also showed significantly decreased levels of BubR1 at 12 months (F1:6 = 51.2, *P* < 0.001 vs. 1 month age). In addition, significantly lower level of BubR1 was observed in SAMP8 than SAMR1 mice at 12 months (F1:6 = 30.2, *P* = 0.002, Fig. 7a, c). By contrast, an increased level of p19 was only observed in SAMP8 mice at 6 months (F1:6 = 31.2, *P* = 0.001 vs. 1 month age; F1:6 = 27, *P* = 0.002 vs. SAMR1 of the same age, Fig. 7a, c). Accordingly, 6-month-old SAMP8 mouse cochleae showed increased activity of SA-β-gal mainly in the spiral ganglion neurons, OHCs, and IHCs (Fig. 7e, h-i, m-o) when compared with SAMR1 mice of the same age (Fig. 7d, f, g, j–l).

#### Correlation Between Molecular Events and Hearing Loss

**Fig. 8:**
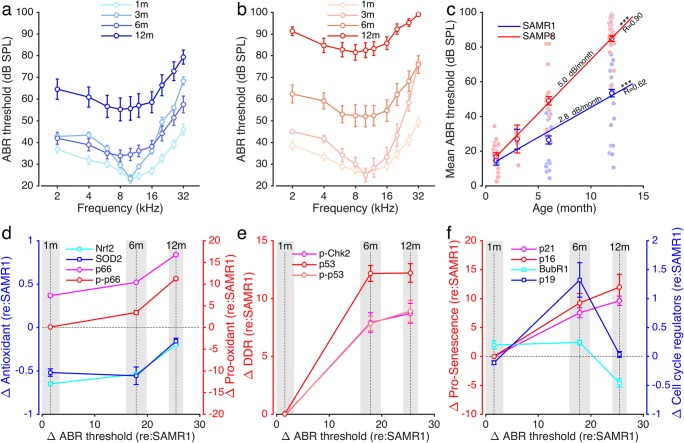
Correlation between molecular events and the hearing loss. **a**, **b** Age-related ABR thresholds in SAMR1 (blue plot) (**a**) and in SAMP8 mice (red plot) (**b**) at 1, 3, 6, and 12 months (*n* = 14 animals per age and per strain). Data are expressed as mean ± SEM. Note the severe elevation in threshold in the SAMP8 mice. **c** The mean ABR threshold from 2 to 32 kHz derived from **a** and **b**. The mean ABR thresholds for each mouse and each time point were calculated for both strains. These values were then used to perform a linear regression allowing the measurement of threshold elevation per month and the calculus of Pearson correlation coefficient. **d**–**f** Time course of hearing loss relating to protein changes in the SAMP8 mice. The *X*-axis represents the mean difference (Δ) in ABR thresholds between SAMP8 and SAMR1 mice, and the gray area represents the SEM of these differences. The left *Y*-axis in **d** represents the delta (Δ) in mean quantities of antioxidants (Nrf2, light blue plot and SOD2 blue plot) observed in SAMP8 mice relative to in SAMR1 mice. The right *Y*-axis in **d** represents the delta (Δ) in mean quantities of pro-oxidants (p66^Shc^, pink plot and p-p66^Shc^, red plot) observed in SAMP8 mice relative to that in SAMR1 mice. The left *Y*-axis in **e** and **f** represents the delta (Δ) in mean quantities of DNA damage response proteins (p-Chk2, pink plot; p53, red plot; and p-p53, light red plot in **e**) and of senescence markers (p21, pink plot and p16, red plot, in **f**). The right *Y*-axis in **f** represents the delta (Δ) in mean quantities of the mitotic checkpoint protein BubR1 (light blue plot in **f**) and of the cell cycle regulator p19 (blue plot in **f**) observed in SAMP8 mice relative to that in SAMR1 mice. The mean quantities of proteins were derived from Figs. 6 and 7

The ABR threshold showed no significant difference between SAMP8 and SAMR1 mice at 1 month (Fig. 8a–c). In contrast, both strains of mice showed an age-related hearing loss after 1 month of age but was twice as severe in SAMP8 (5 dB/month, *R* = 0.9, *P* < 0.001) than in SAMR1 mice (2.8 dB/month, *R* = 0.62, *P* < 0.001, Fig. 8c). To facilitate the comparison between the two strains of mice, we expressed all the functional data obtained in SAMP8 with respect to those in SAMR1. We then expressed the molecular events as a function of hearing threshold shift with respect to the control SAMR1. When the molecular event was close to zero, hearing loss was considered mainly due to age and independent of the strain of mouse. In contrast, a positive or negative shift indicated a correlation between molecular event and the phenotype of SAMP8 (i.e., accelerated hearing loss).

The SAMP8 strain displayed a deficit in antioxidant proteins Nfr2 and SOD2 and an excess of the pro-oxidant molecule p66^Shc^ at all ages. The levels of pro-oxidant molecules p66^Shc^ and p-p66^Shc^ increased concomitantly with the degradation of hearing in SAMP8, while the deficit in antioxidants remained relatively stable until 6 months of age (Fig. 8d). The differences between the strains in terms of levels of the antioxidant molecules Nrf2 and SOD2 decreased at 12 months of age (Fig. 8d), probably due to the age-related repression of these molecules in both strains. While both strains displayed comparable levels of DDR proteins (p-Ch2, p53, p-p53) at 1 month, only SAMP8 mice thereafter showed a dramatic increase in levels to reach a plateau at 6 months (Fig. 8e). Finally, prosenescence proteins (p21, p16) increased continually with age in SAMP8 mice (Fig. 8f). By contrast, the mitotic checkpoint protein BubR1 and the cell cycle regulator p19 remained close to zero, except at 6 months where p19 had increased before falling back to zero at 12 months with a net result of no significant difference between strains at 12 months (Fig. 8f).

Collectively, these in vivo data support our in vitro results indicating that an early increase in the level of ROS triggers the activation of DNA damage responses and premature occurrence of senescence-like state in postmitotic cochlear cells and an accelerated hearing loss in SAMP8 mice.

#### EUK-207 Slows Down Age-Related Hearing Loss in SAMP8 Mice

**Fig. 9:**
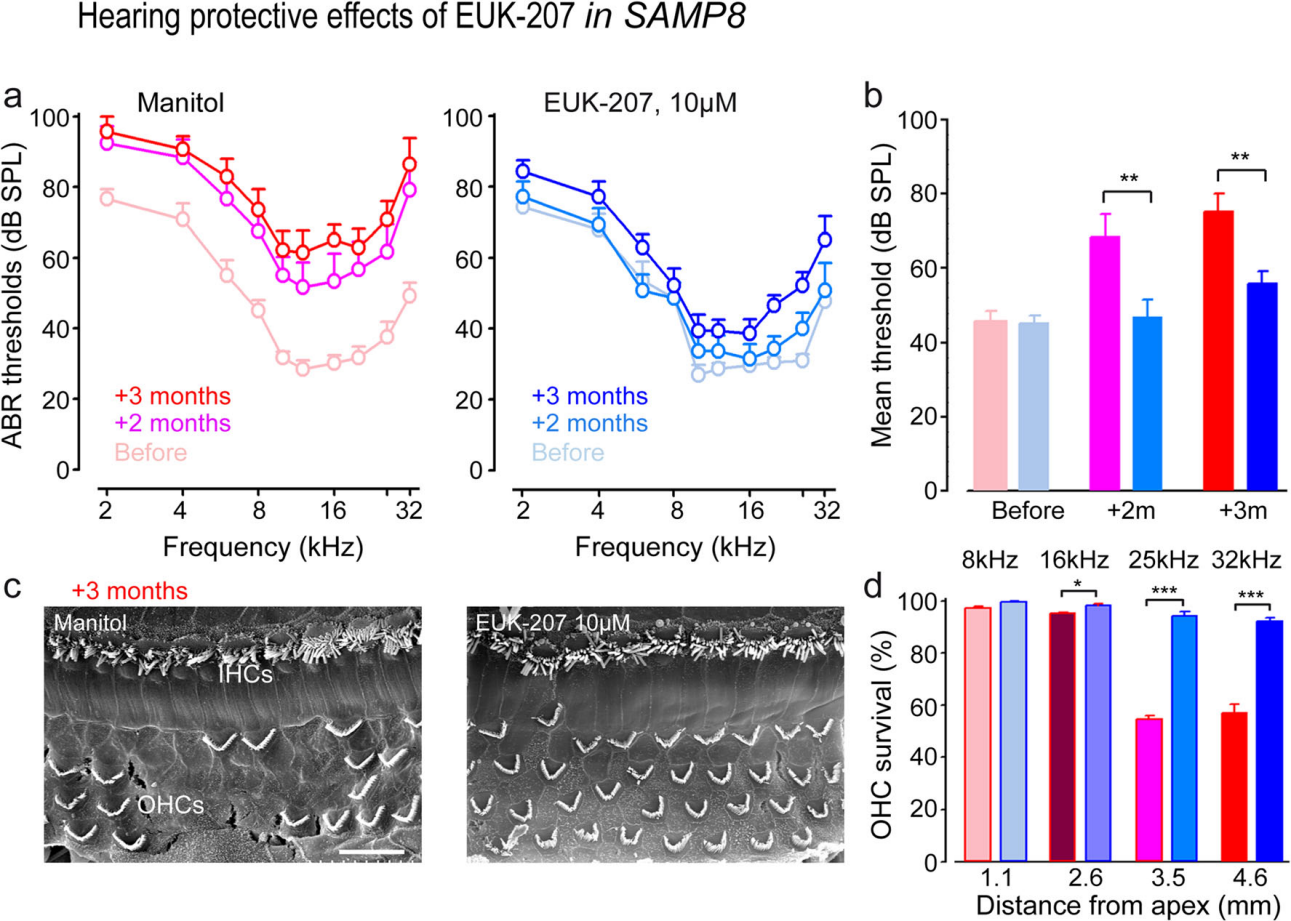
Pharmacological mitigation of ROS prevents loss of hearing and hair cells in adult mice. **a** ABR thresholds recorded before (pale red plot) and after 2 months (pink plot) and 3 months (red plot) of mannitol treatment, or before (pale blue plot) and after 2 months (azure plot) and 3 months (blue plot) of EUK-207 treatment. **b** Mean ABR threshold from 2 to 32 kHz derived from **a**. Data are expressed as mean ± SEM (*n* = 10 per group). One-way ANOVA test was followed by post hoc Tukey’s test (**P ≤ 0.006, ***P ≤ 0.001 vs. mannitol). **c** Representative scanning electron microscopy micrographs showing the basal regions of cochleae from mannitol-treated and EUK-207-treated SAMP8 mice after 3 months of treatment. Scale bar = 15 μm. **d** Cytocochleograms representing the percentage of surviving hair cells in four cochlear regions located at 1.1, 2.6, 3.5, or 4.1 mm from the cochlear apex from mannitol-treated (red bars) and EUK-207-treated (blue bars) SAMP8 mice (*n* = 7 per group). Data are expressed as mean ± SEM. One-way ANOVA test was followed by post hoc Tukey’s test (*P ≤ 0.028, ***P ≤ 0.001 vs. mannitol-treated group)

With a view to developing pharmacological therapies in vivo, we began treatment of groups of SAMP8 mice from 6 months of age with either EUK-207 dissolved in mannitol or with mannitol alone as a control, delivered by subcutaneously implanted osmotic pumps (see “Material and Methods” section). In contrast with mannitol-treated control SAMP8 mice, 2- and 3-month treatments with EUK-207 significantly prevented the age-related ABR threshold increase (Fig. 9a, b) (2 months: F1:13 = 11, *P* = 0.006, 3 months: F1:15 = 14.4, *P* = 0.002). The mean thresholds from EUK-207-treated and mannitol-treated SAMP8 mice were 55.7 ± 3.5 and 75.1 ± 4.7 dB, respectively, at the end of the 3-month treatment (Fig. 9a, b). Consistent with this finding, hair cell counting using scanning electron microscopy at the end of 3 months of treatment showed greater OHC survival in the basal region of EUK-207-treated mice (16 kHz region: F1:9 = 7.2, *P* = 0.028; 25 kHz: F1:9 = 657.6, *P* < 0.001; 32 kHz: F1:10 = 67.6, *P* < 0.001, Fig. 9c, d). OHC survival rates in EUK-207-treated mice and mannitol-treated mice were 92.2 ± 1.3 and 57.12 ± 3.44%, respectively, at a frequency of 32 kHz (Fig. 9c, d). Altogether, these results indicate that the mitigation of excessive ROS with EUK-207 prevented accelerated age-related hearing loss and sensory hair cell loss in SAMP8 mice.

## Discussion

ARHL is the third most prevalent chronic medical condition affecting older adults [1]. Although the etiology of ARHL remains unclear, oxidative stress is proposed as one of the most important risk factors [4, 6]. Consistent with the results obtained from other cell types [30, 31], cochlear sensory hair cells displayed dose-dependent cytotoxic effects in cochlear explants exposed to H_2_O_2_. In order to examine the potential ROS-related mechanisms leading to cochlear aging, we set the level of ROS to resemble as closely as possible the in vivo conditions. Even at such low concentrations, H_2_O_2_ led to oxidative stress–related events in cochlear tissues 3 days after exposure. Considering the common link between oxidative stress and autophagy [34], we assessed autophagy induction by monitoring autophagosome formation and autophagic flux. As suspected, H_2_O_2_-induced oxidative stress initiated autophagy, as illustrated by the activation or increased expression of several autophagy-related proteins, such as Beclin 1, LC3-II, Rab7, and p62. The increased level of the autophagic substrate p62 in particular indicated an impaired autophagic flux [49]. Collectively, these results demonstrate that at low concentrations (below EC_50_), H_2_O_2_ can induce oxidative stress and trigger an impaired autophagic response, and they thereby validate our in vitro model of oxidative stress.

Unlike the necrosis or apoptosis observed in cells subjected to cytotoxic doses of H_2_O_2_, we have here discovered that exposure to low concentrations of H_2_O_2_ slightly alters cochlear cell viability within 3 days after exposure but importantly induces DNA damage, mainly in sensory hair cells. These results are consistent with our previous study in which genotoxic stress was induced by cisplatin, a DNA-damaging anticancer drug [12]. In the present study, H_2_O_2_ exposure induced DNA damage responses within cochlear cells, as shown by significant increases in DDB2, a protein involved in nucleotide excision repair [43], p-Chk2, and p53. Depending on the severity of the DNA damage, the cell microenvironment, and the cell type, activated p53 can orchestrate different cellular outcomes such as repair of DNA damage, apoptosis, or senescence [50].

DNA damage and DDR are known to be involved in the induction of replicative senescence and DNA damaging agent-induced premature senescence in cycling cells [51, 52]. Here, we have demonstrated for the first time that the ROS-induced DDR coincided with the premature occurrence of senescence-like features in postmitotic cochlear cells, as shown by increased levels of p21, a substrate of p53, and a necessary signal transducer between DDR and senescence in neurons [53]. Consistent with the increase in p21, H_2_O_2_-exposed cochleae displayed high levels of p38 and phospho-p38. These latter two proteins can be activated by p21 and may be implicated in senescence-associated secretory phenotypes [54]. Together with increased activity of SA-β-gal following H_2_O_2_ exposure, our results indicate that the activation of the p53–p21 pathway might mediate the low concentration H_2_O_2_-induced senescence-like phenotype in postmitotic cochlear cells in vitro. To shed more light on these postmitotic cochlear cell senescence-like phenotypes, we quantified the levels of p16, named also p16^INK4A^, which can be induced independently of DDR and p53. Above a certain threshold, p16 blocks G1–S progression within cycling cells [55] and may act as an effector of senescence in cultured cells [56]. However, our H_2_O_2_-exposed cochleae exhibited no significantly increased levels of p16 arguing against the engagement of the p16 pathway in our in vitro conditions.

We also assessed the level of the mitotic checkpoint protein BubR1, the level of which decreases with age in various mouse tissues [57]. BubR1 insufficiency is associated with the occurrence of multiple progeroid and aging-associated phenotypes in mice [58] and in children with mosaic variegated aneuploidy syndrome [59]. Consistent with these reports, we found a significantly decreased expression of BubR1 after H_2_O_2_ challenge suggesting its implication in ROS-induced premature cochlear cell senescence-like phenotypes. We also found a significantly decreased expression of the cell cycle regulator p19 in H_2_O_2_-exposed cochleae. This protein likely plays a role in the attenuation of senescence and aging in BubR1-insufficient mice [60], yet the precise relationship between its decreased level and increased cochlear cell senescence-like phenotypes after ROS challenge requires further in-depth investigation.

To probe the impact of ROS mitigation on H_2_O_2_-induced DNA damage and senescence-like phenotypes, we tested a synthetic SOD/catalase mimetic, EUK-207. This molecule possesses both SOD and catalase activity and scavenges ROS generated in the cytoplasm or organelles, including the mitochondria [17]. In addition, EUK-207 suppresses oxidative modifications of proteins, lipids, and nucleic acids [61]. Here, we found that cochleae treated with EUK-207, alone or in combination with H_2_O_2_, displayed increased levels of FOXO3a and Nrf2. These two transcription factors have been shown to positively regulate cellular resistance to oxidative stress [47, 62]. Increasing their levels of expression would be expected therefore to account for the observed protective effect of EUK-207 against H_2_O_2_-induced DNA damage and senescence-like phenotype in cochlear cells in culture.

To extend these in vitro data to the system level, we used SAMP8 mice. This strain of mouse develops a premature ARHL and early onset cochlear cell degeneration [4]. Here, we showed that, compared to control, SAMP8 cochleae displayed an early increased oxidative stress (1 month of age), highlighted by significantly lower levels of both Nrf2, a main regulator of cellular resistance to oxidants, and the antioxidant enzyme SOD2, concomitant with a large increase in the levels of p66^Shc^ and its phosphorylated form. These two latter proteins are involved in downregulating the synthesis of antioxidant enzymes and the generation of ROS in the mitochondria [63]. These in vivo data indicate that the events related to oxidative stress occur early (1 month of age), before the onset of hearing loss in SAMP8 mice (3 months) [4]. Here, we also found significantly higher levels of phosphorylated Beclin 1, Rab7, and p62 in SAMP8 mice during aging compared with SAMR1. Together with our previous study demonstrating the upregulation of LC3-II with concomitant accumulation of lipofuscin in SAMP8 mouse cochleae, we suggest an increased autophagy with impaired and/or insufficient capacity for autophagy flux in SAMP8 mice. These results are consistent with some recent studies showing that autophagy plays an important role in maintaining adult hearing in response to auditory stress or during aging. Indeed, activation of autophagy by rapamycin diminished noise- [64] or cisplatin-induced [65] hair cell and hearing loss. Moreover, the weaker autophagy gene expression in 1-year-old cochleae of Igf1^−/−^ mice coincides with age-related hearing loss [66, 67].

Consistent with our in vitro results, the SAMP8 mice displayed activation of the Chk2–p53 pathway from 6 months of age. From the same age, they also displayed similar senescence signatures, characterized by decreased levels of BubR1 and increased levels of p21 and p16, together with increased activity of SA-β-gal in the region of spiral ganglion neurons, OHCs, and IHCs of SAMP8 mice. These discoveries are consistent with recent studies in which activation of DNA damage responses and the appearance of certain cell senescence markers were implicated in postmitotic neuron senescence [68, 69]. Based on the significant correlation between early increased oxidative stress together with later accumulation of DDR and senescence-like features, and severe hearing loss in SAMP8 mice, we suggest that oxidative stress may be one of the main culprits behind age-related cochlear cell degeneration and auditory function decline.

Having established the causal relationship between ROS and age-related cochlear cell degeneration, we set out to test the efficiency of systemically administering EUK-207 to mitigate the pathological effects of uncontrolled ROS production on hearing function. Our results reveal that systemic treatment with EUK-207 effectively prevented or slowed down ARHL in SAMP8 mice. These results are consistent with previous studies showing successful suppression of age-related cognitive impairment in mice with EUK-207 [15, 16]. They also represent a proof-of-concept that pharmacological scavenging of superoxide and hydrogen peroxide can mitigate ARHL in SAMP8 mice. They thereby provide a strong rationale for the clinical development of EUK-207 for use in the prevention or slowing down of ARHL in humans.

## Conclusion

The in vitro and in vivo data presented here provide evidence that (i) ROS is one of the main culprits behind age-related sensory hair cell degeneration, (ii) ROS-induced DDR driving senescence-like features may account for premature cochlear aging, and (iii) pharmacological scavenging of superoxide and hydrogen peroxide can mitigate ARHL in SAMP8 mice.

## Abbreviations

*ARHL*: age-related hearing loss
*Cat*: catalase
*DDB2*: DNA damage–binding protein 2
*DDR*: DNA damage response
*IHCs*: inner hair cells
*Nrf2*: nuclear factor erythroid 2–related factor 2
*OHCs*: outer hair cells
*p-Chk1*: checkpoint kinase 1
*p-Chk2*: checkpoint kinase 2
*ROS*: reactive oxygen species
*SAMP8*: senescence-accelerated prone 8 mice
*SAMR1*: senescence-accelerated resistant 1 mice
*SOD*: superoxide dismutase
